# Risk Factors and Treatment Trends for Onychomycosis: A Case–Control Study of Onychomycosis Patients in the All of Us Research Program

**DOI:** 10.3390/jof9070712

**Published:** 2023-06-29

**Authors:** Samantha Jo Albucker, Julianne M. Falotico, Zi-Ning Choo, Justin T. Matushansky, Shari R. Lipner

**Affiliations:** 1 Tulane University School of Medicine, New Orleans, LA 70112, USA; salbucke@tulane.edu; 2 Renaissance School of Medicine at Stony Brook University, Stony Brook, NY 11794, USA; julianne.falotico@stonybrookmedicine.edu; 3 Weill Cornell Medical College, New York, NY 10021, USA; zic2001@med.cornell.edu; 4 Columbia University, New York, NY 10027, USA; matushanskyjustin23m@gmail.com; 5 Department of Dermatology, Weill Cornell Medicine, New York, NY 10021, USA

**Keywords:** antifungals, comorbidities, fungus, nail disease, nail infection, nail dystrophy, onychomycosis, procedures

## Abstract

**Introda significant**: Onychomycosis is the most common nail disorder seen in clinical practice, and it may have significant impact on patient quality of life. Understanding risk factors for onychomycosis may help to devise screening and treatment guidelines for populations that are more susceptible to this infection. Using a national database, we aimed to explore associations between onychomycosis and age, sex, and underlying medical conditions, as well as to examine current onychomycosis treatment trends. **Materials and Methods**: We performed a nested, matched, case–control study of patients in the All of Us database aged ≥ 18 years (6 May 2018–1 January 2022). Onychomycosis cases were identified using International Classification of Diseases (ICD) and Systematized Nomenclature of Medicine (SNOMED) diagnostic codes (ICD-9 110.1, ICD-10 B35.1, SNOMED 414941008). Demographic information (i.e., age, sex, and race), treatments, and co-diagnoses for onychomycosis patients and case–controls were recorded. Wald’s test applied to multivariate logistic regression was used to calculate odds ratios and *p*-values between onychomycosis and co-diagnoses. Additionally, 95% confidence intervals were calculated with a proportion test. **Results**: We included 15,760 onychomycosis patients and 47,280 matched controls. The mean age of onychomycosis patients was 64.9 years, with 54.2% female, 52.8% Non-Hispanic White, 23.0% Black, 17.8% Hispanic, and 6.3% other, which was similar to controls. Patients with onychomycosis vs. controls were more likely to have a co-diagnosis of obesity (46.4%, OR 2.59 [2.49–2.69]), tinea pedis (21.5%, OR 10.9 [10.1–11.6]), peripheral vascular disease (PVD) (14.4%, OR 3.04 [2.86–3.24]), venous insufficiency (13.4%, OR 3.38 [3.15–3.59]), venous varices (5.6%, OR 2.71 [2.47–2.97]), diabetes mellitus (5.6%, OR 3.28 [2.98–3.61]), and human immunodeficiency virus (HIV) (3.5%, OR 1.8 [1.61–2.00]) (*p* < 0.05, all). The most frequently prescribed oral and topical medications were terbinafine (20.9%) and ciclopirox (12.4%), respectively. The most common therapeutic procedure performed was debridement (19.3%). Over the study period, ciclopirox prescriptions (Spearman correlation 0.182, *p* = 0.0361) and fluconazole prescriptions increased (Spearman correlation 0.665, *p* = 2.44 × 10^−4^), and griseofulvin (Spearman correlation −0.557, *p* = 0.0131) and itraconazole prescriptions decreased (Spearman correlation −0.681, *p* = 3.32 × 10^−6^). **Conclusions**: Our study demonstrated that age, obesity, tinea pedis, PVD, venous insufficiency, diabetes mellitus, and HIV were significant risk factors for onychomycosis. In addition, the most frequent oral and topical onychomycosis medications prescribed were terbinafine and ciclopirox, likely reflective of efficacy and cost considerations. Identifying and managing these risk factors is essential to preventing onychomycosis’ primary infections and recurrences and improving treatment efficacy.

## 1. Introduction

With a worldwide prevalence of 5.5%, onychomycosis is the most common nail disorder seen in clinical practice [1]. A majority of onychomycosis cases in the United States are caused by dermatophytes, including *Trichophyton rubrum* and *Trichophyton mentagrophytes* (60–70%), followed by non-dermatophyte molds, including *Scopulariopsis brevicaulis*, *Aspergillus* spp., *Acremonium*, *Fusarium* spp., *Alternaria* alternate, and *Neoscytalidium* (20%), as well as yeasts, including *Candida* spp. (10–20%) [1]. Some risk factors for nail fungal infections include older age, diabetes mellitus, immunosuppression, tinea pedis, hyperhidrosis, obesity, and household contacts with fungal infections [1].

While onychomycosis is a benign and treatable nail condition, it can have a considerable impact on patients’ everyday lives. In a systematic review analyzing the impact of onychomycosis on quality of life (QoL), onychomycosis resulted in physical impairment, decreased functionality, pain or discomfort, and social embarrassment, with psychological and psychosocial impact as high as 92% in affected patients [2]. Moreover, in another systematic review of thirty onychomycosis studies, quality of life scores improved from baseline with all treatment types, with greater improvements reported with oral treatments in comparison to topical treatments [3]. Hence, onychomycosis is not just an aesthetic problem, and appropriate diagnosis and management can significantly improve QoL.

A previous cross-sectional study utilized the All of Us database to study the prevalence of onychomycosis in underrepresented groups (30 May 2017 to 1 April 2021) [4]. The authors reported higher adjusted odds of onychomycosis in patients ages 75 years or older (OR, 1.85; 95% CI, 1.75–1.96), sexual orientation/gender identity status (i.e., LGBTQIA+) (OR, 1.12; 95% CI, 1.05–1.19), less than a high school education (OR, 1.05; 95% CI, 0.98–1.13), annual income less than or equal to USD 35,000 (OR, 1.04; 95% CI, 0.99–1.09), and physical disability (OR, 1.14; 95% CI, 1.08–1.20) [4]. This study did not evaluate onychomycosis risk factors, including medical comorbidities or treatment trends. 

Therefore, we aimed to explore the relationships between onychomycosis and age, sex, race, and underlying medical conditions using the national database All of Us. Understanding risk factors for onychomycosis may inform screening guidelines for populations that are typically more susceptible to infection, as well as treatment guidelines. Furthermore, our investigation sought to analyze the current treatment trends for onychomycosis. We examined the utilization of oral antifungal treatments, topical antifungal treatments, and various procedures in the management of this condition. The understanding gained from analyzing treatment patterns may provide valuable information regarding the effectiveness and preferences for different therapeutic approaches. 

## 2. Materials and Methods

We performed a comprehensive nested, matched, case–control study utilizing the data available in the All of Us database. The All of Us database includes patients aged 18 years and older recruited within the period of 6 May 2018 to 1 January 2022. Individuals can volunteer to participate in the All of Us program either directly or indirectly (through their healthcare provider organizations). In the All of Us database, volunteers can complete health surveys, consent to the sharing of their electronic health records (EHRs), and may provide biological samples. The health records are then transferred to the All of Us platform, and the information is deidentified. The data are then made accessible for users to utilize for respective research purposes.

In this study, onychomycosis cases were identified in the All of Us database using the International Classification of Diseases (ICD) and the Systematized Nomenclature of Medicine (SNOMED) diagnostic codes. Specifically, we employed the ICD-9 code 110.1, the ICD-10 code B35.1, and the SNOMED code 414941008. By utilizing these codes, we were able to exclusively identify individuals with confirmed cases of onychomycosis within the All of Us database.

To gain a comprehensive understanding of the population affected by onychomycosis, we collected demographic information for both the onychomycosis patients and carefully matched controls. This information included age, sex, and race, which allowed us to analyze potential associations between these factors and the development of onychomycosis. In our study, we also focused on several co-diagnoses that are known to have potential associations with onychomycosis. These co-diagnoses included obesity (All of Us concept code 433736), tinea pedis (All of Us concept code 133141), Peripheral vascular disease (PVD) (All of Us concept code 3654996), venous insufficiency (All of Us concept code 321596), venous varices (All of Us concept code 312349), diabetes mellitus (All of Us concept code 201820), human immunodeficiency virus (HIV) (All of Us concept code 439727), immunosuppression (All of Us concept code 4242843), and hyperhidrosis (All of Us concept code 138565). 

In addition to analyzing the co-diagnoses, we also investigated the usage of specific oral and topical medications commonly prescribed for the treatment of onychomycosis. Oral medications included terbinafine (All of Us concept codes 1741402, 1741309, 40171255, and 19006863), fluconazole (All of Us concept codes 1754996, 1754994, 1909003, and 19034537), griseofulvin (All of Us concept codes 1763846, 19077740, 1763779), and itraconazole (All of Us concept codes 19078728 and 1703653). Topical medications included ciclopirox (All of Us concept codes 19075343, 19075343, 950133, and 950098), efinaconazole (All of Us concept codes 45775087, 45775083, and 45775080), and tavaborole (All of Us concept codes 45776544, 45776540, and 45776537). Furthermore, we explored various procedures related to onychomycosis treatment that were documented in the All of Us database. These procedures included the debridement of nail(s) (All of Us concept codes 2102077, 2102076, and 2006538), nail avulsion (including either partial or complete) (All of Us concept codes 2102078, 2102079, and 2722193), nail removal (All of Us concept codes 4295216), and the trimming of nail(s) (All of Us concept code 2617229) and were also recorded. 

To analyze the collected data, we employed several advanced statistical techniques. Wald’s test, applied in the context of multivariate logistic regression, allowed us to calculate odds ratios (ORs) and associated *p*-values values between onychomycosis and demographic information, onychomycosis and co-diagnoses, and onychomycosis and various treatments. These measures helped us to evaluate the strength and significance of the relationship between onychomycosis and the identified demographic information, co-diagnoses, medications, and procedures. Additionally, we calculated 95% confidence intervals (Cis) to assess the precision of our estimates and to provide a range within which the true population values are likely to fall. In order to account for multiple hypothesis testing, we applied the Benjamini–Hochberg procedure, which helped control the false discovery rate (FDR). By adjusting the *p*-values, this procedure helped us minimize the risk of falsely identifying significant associations. We considered any condition corresponding to an FDR less than 0.1 as statistically significant, ensuring that our findings were reliable.

## 3. Results

**Figure 1:**
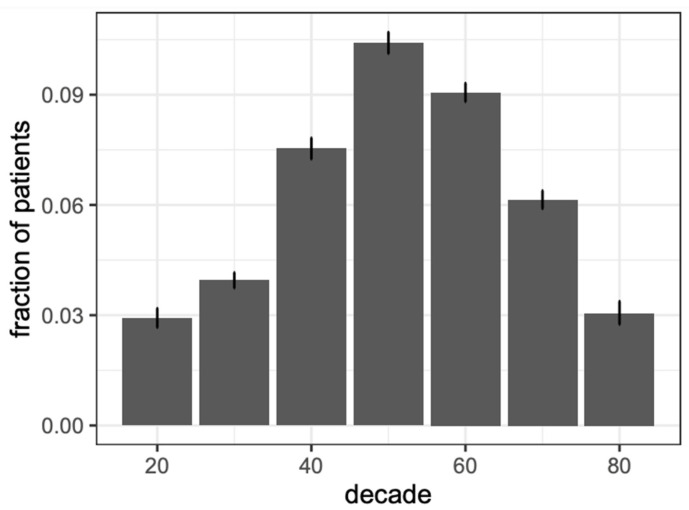
Prevalence of patients with an onychomycosis diagnosis grouped by age at the time of diagnosis. Fraction of patient refers to number of onychomycosis patients/number of patients with EHR data of the same age.

A total of 15,760 onychomycosis patients and 47,280 matched controls were analyzed (Table 1). The mean age of onychomycosis patients was 64.9 years old (SD 12.9), with 54.2% females, 52.8% Non-Hispanic Whites, 23.0% Blacks, 17.8% Hispanic, and 6.3% other, with similar demographics to control patients. The mean age by sex for females was 63.7 years old (SD 13.0), males was 66.2 years old (SD 12.6), and other was 66.3 years old (SD 12.2). The age cohort with the highest frequency of onychomycosis, which was a fraction determined by the number of onychomycosis patients per the number of patients with EHR data of the same age, was 50 to 59 years (fraction = 0.105, *n* = 4371, 95% CI [0.102–0.108]), followed by 60 to 69 (fraction = 0.090, *n* = 4493, 95% CI [0.088–0.093]), 40 to 49 (fraction = 0.075, *n* = 2424, 95% CI [0.073–0.078]), 70 to 79 (fraction = 0.060, *n* = 2255, 95% CI [0.058–0.063]), 20 to 29 (fraction = 0.031, *n* = 477, 95% CI [0.028–0.034]), and 80 to 89 (fraction = 0.029, *n* = 357, 95% CI [0.026–0.032]) (Table 2). Onychomycosis prevalence increased between the ages of 20 and 49 and decreased between the ages of 60 and 89 years (Figure 1). 

Patients with a diagnosis of onychomycosis in comparison to controls were more likely to have a co-diagnosis of obesity (46.4%, OR 2.59 [2.49–2.69]), tinea pedis (21.5%, OR 10.9 [10.1–11.6]), PVD (14.4%, OR 3.04 [2.86–3.24]), venous insufficiency (13.4%, OR 3.38 [3.15–3.59]), venous varices (5.6%, OR 2.71 [2.47–2.97]), diabetes mellitus (5.6%, OR 3.28 [2.98–3.61]), and HIV (3.5%, OR 1.8 [1.61–2.00]) (*p* < 0.05, all). 

The most frequently prescribed systemic medications were terbinafine (20.9%, 95% CI [20.2–21.5]) and fluconazole (17.1%, 95% CI [16.6–17.8]), whereas griseofulvin (1.09%, 95% CI [0.93–1.26]) and itraconazole (1.05%, 95% CI [0.91–1.24]) were least commonly prescribed. The most frequent topical medication prescribed was ciclopirox (12.4%, 95% CI [11.9–13.0]), whereas efinaconazole (2.6%, 95% CI [2.31–2.81]) and tavaborole (0.3%, 95% CI [0.26–0.45]) were less commonly prescribed. The most commonly performed procedure was debridement of nail(s) (19.3%, 95% CI [18.7–19.9]), followed by nail avulsion or nail removal (4.4%, 95% CI [4.04–4.70]) and trimming of nail(s) (2.9%, 95% CI [2.69–3.22]). 

**Figure 2:**
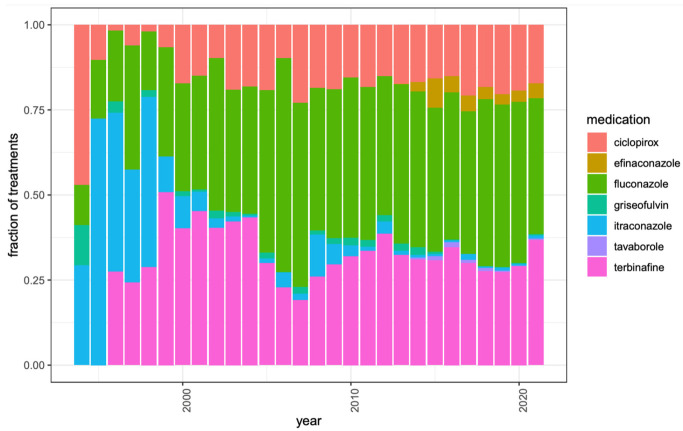
Antifungal medication prescription trends over time for patients with a diagnosis of onychomycosis in the All of Us research program. The height of each bar represents the number of onychomycosis patients prescribed that antifungal medication divided by the number of onychomycosis patients prescribed any antifungal medication that year. Only years with at least twenty onychomycosis cases in All of Us are shown.

Over time, there was an increase in ciclopirox prescriptions (Spearman correlation 0.182, *p* = 0.0361) and an increase in fluconazole prescriptions (Spearman correlation 0.665, *p* = 2.44 × 10^−4^). In addition, there was a decrease in griseofulvin prescriptions (Spearman correlation −0.557, *p* = 0.0131) and a decrease in itraconazole prescriptions (Spearman correlation −0.681, *p* = 3.32 × 10^−6^) (Table 3, Figure 2). 

## 4. Conclusions

Our study demonstrated that age, obesity, tinea pedis, PVD, venous insufficiency, diabetes mellitus, and HIV were all significant risk factors for developing onychomycosis. There were no significant associations with either hyperhidrosis or immunodeficiency with onychomycosis. In addition, the most frequent oral and topical onychomycosis medications prescribed were terbinafine and ciclopirox, respectively, and the most common therapeutic procedure performed was the debridement of nail(s). Over time, ciclopirox prescriptions and fluconazole prescriptions increased, and griseofulvin and itraconazole prescriptions have decreased.

In our onychomycosis cohort, the mean age was 64.9 years old, and the group with the highest frequency of onychomycosis was 50 to 59 years. Similarly, in a survey-based study of 254 patients attending a vascular clinic, 22.4% of patients were diagnosed with onychomycosis, and onychomycosis was associated with increasing age (risk odds ratio (ROR) 1.05, *p* = 0.002) [5]. Another cross-sectional study of 86 patients (44 onychomycosis patients and 42 controls) similarly identified as a significant risk factor for onychomycosis age (OR 1.11, 95% CI [1.03–1.19]) [6]. In a post hoc analysis of 1062 patients with onychomycosis, cure rates were lower in older patients (i.e., greater than 65 years old) compared to younger patients (i.e., less than 40 years old) (16.7% vs. 23.4%) [7]. While our study also showed that onychomycosis prevalence decreased between the ages of 60 and 89 years, this may reflect the fact that some older adults may not prioritize treating nail diseases because management of other comorbidities may take precedent. Older adults may have poor peripheral circulation, prolonged exposure to fungi, recurring trauma to the nails, immunodeficiency, and slower rates of nail growth, all of which might contribute to their significant risk of onychomycosis [7]. Patients with limited dexterity or physical conditions may find it exceedingly challenging to reach their feet for the purpose of applying topical antifungal medications, thus further complicating the treatment process. Moreover, patients with impaired vision and reduced dexterity may face additional obstacles in effectively carrying out the treatment plan. Lastly, the intricate interplay of drug–drug interactions and polypharmacy may influence physicians’ choices of treatment in older adults [7]. 

Our study also demonstrated that patients with a diagnosis of onychomycosis compared to matched case–controls were more likely to have a co-diagnosis of obesity. Similarly, in an observational cross-sectional study of 500 patients, with patients by grouped by BMI (control group: less than 25, overweight group: 25 to 29.9, obese group: greater than 30), tinea pedis (48.0%) and onychomycosis (36.4%) were relatively common in the obese group [8]. Tinea pedis and onychomycosis were more prevalent in the obese group compared to the controls and in the overweight group compared to the controls (<0.05, all) [8]. In a post hoc analysis of 1233 onychomycosis patients treated with efinaconazole topical solution 10%, complete cure rates at week 52 were 15.9% in obese patients, compared to 22.0% in non-obese patients (*p* = 0.05) [7]. This decreased efficacy may have been due to comorbidities common to patients with obesity that also contribute to onychomycosis risk, such as medication noncompliance (i.e., difficulty reaching one’s feet in order to apply topical treatments) or poor overall nail hygiene (i.e., difficulty showering) [7].

Our study also demonstrated that patients with a diagnosis of onychomycosis compared with matched case–controls were more likely to have a co-diagnosis of tinea pedis. Similarly, in a prospective survey study of 2761 patients, 42.8% had toenail onychomycosis and fungal skin infections, with tinea pedis being the most common (33.8%) [9]. Additionally, in a multicenter, double blind, vehicle-controlled study evaluating the safety and efficacy of efinaconazole topical solution 10% in 1655 patients diagnosed with onychomycosis, 21.3% of patients had coexisting interdigital tinea pedis [10]. Moreover, complete cure rates with efinaconazole were higher in patients diagnosed with onychomycosis cotreating tinea pedis (29.4%) than in patients diagnosed with onychomycosis who were not cotreating tinea pedis (16.1%) (*p* = 0.003 and 0.045, respectively) [10]. Daily treatment with topical antifungals to the feet may prevent primary onychomycosis infections since tinea pedis is typically the precursor of onychomycosis. Daily antifungal treatment may also decrease the rate of recurrence by preventing reinfection [11]. 

Our study also supports the association between onychomycosis and PAD. Similarly, in a cross-sectional study of 254 patients attending a vascular clinic, 22.4% had onychomycosis with PAD as a risk factor (ROR 4.8, *p* = 0.02) [6]. In another cross-sectional study of 86 patients, the prevalence of PAD was higher in patients with a diagnosis of onychomycosis than in those without a diagnosis of onychomycosis (31.8% vs. 4.8%) (*p* = 0.001), and a multiple logistic analysis identified PAD as a risk factor for onychomycosis (OR 9.85, 95% CI [1.37–70.72]) [7]. Mean Ankle/Brachial Index (ABI) values for both legs were lower in onychomycosis patients (0.998) compared to controls (1.133) (*p* = 0.001), and the hazard ratios for total and cardiovascular mortality consistently increased with decreasing ABI (ABI 0.91–1.00; men: 1.61, 1.68; women: 1.52, 1.84, respectively) for levels of ABI less than 1.00 [7]. While it may not be practical or feasible to record ABI on all patients with onychomycosis, particularly in dermatology or podiatry practices, it is still an important to keep in mind the association between onychomycosis and PAD. Moreover, the presence of onychomycosis could be an indication of a deterioration of the ABI in persons with PAD; hence, onychomycosis could provide a simple prognosticator of PAD, and it may be beneficial to screen patients diagnosed with onychomycosis for cardiovascular risk factors. 

We found that onychomycosis was associated with co-diagnosis of venous insufficiency. Similarly, in a case–control study of 70 patients (33 onychomycosis patients and 37 controls), in which lower extremity veins were examined with Doppler ultrasound, onychomycosis patients had venous insufficiency more frequently than the control group (42.4% and 10.8%, respectively; *p* = 0.003) [12]. Although all of the patients with onychomycosis had both feet involved, reflux was bilateral in only 48.8% of patients, questioning the mechanism of the association. In another case–control study of 81 patients, venous insufficiency was detected more frequently in patients diagnosed with onychomycosis than in the control group (35.7% and 15.4%, respectively; *p* = 0.037) [13]. Therefore, physicians should consider referral for venous Doppler ultrasound exams for patients diagnosed with onychomycosis to rule out coexisting venous insufficiency.

We showed that patients with a diagnosis of onychomycosis compared to matched case–controls were more likely to have a co-diagnosis of diabetes mellitus. In a multicenter study of 550 patients diagnosed with diabetes, after controlling for age and sex, OR for co-diagnosis of toenail onychomycosis for diabetics was 2.77 times higher than that of healthy controls (95% CI [2.15–3.57]) [14]. In a cross-sectional study of 86 patients, the prevalence of diabetes was significantly higher in patients diagnosed with onychomycosis compared to those without onychomycosis, and multiple logistic analysis identified diabetes as a significant risk factor for onychomycosis (OR 175.11, 95% CI [12.57–2440.32]) [6]. In a case–control study of 75 patients, 53.3% and 46.7% of diabetic patients had onychomycosis and tinea pedis, respectively, with the prevalence of both fungal infections significantly higher than in the control group [15]. Moreover, previous toe amputation was significantly associated with both skin fungal infections and onychomycosis [15]. 

We found that patients with a diagnosis of onychomycosis compared to matched case–controls were more likely to have a co-diagnosis of HIV. Similarly, in a multicenter survey study of 500 patients, the prevalence of onychomycosis in HIV-positive individuals was 23.2% [16]. In a prospective controlled study of 155 HIV-positive patients and 103 HIV-negative control subjects, onychomycosis was present in 30.3% of the HIV-positive patients compared to 12.6% of the HIV-negative controls (*p* = 0.001) [17]. The mean CD4+ cell count was lower in patients diagnosed with onychomycosis compared to those without onychomycosis (0.38 ± 0.30 vs. 0.49 ± 0.32, *p* = 0.03), and the prevalence of onychomycosis increased in advanced stages of HIV disease (21% stage A disease, 30% stage B, 54% stage C, *p* < 0.02) [17]. While onychomycosis was reported in the early HIV stages, more severe involvement (10 to 20 nails, 5 patients) was more common in advanced HIV stages [17]. Onychomycosis in patients diagnosed with HIV is likely related to the level of immunosuppression, and ensuring that patients are well controlled on HIV treatment may decrease the risk of onychomycosis.

Our study found that the most frequently prescribed systemic medications were terbinafine and fluconazole, with fluconazole prescriptions increasing over time. Similarly, in a retrospective observational study of onychomycosis antifungal prescription cost and trends in utilization by dermatologists (2013 to 2018), terbinafine claims experienced an annual average growth rate of 6.7%, and total spending increased by 15.1% [18]. Of note, the cost of the medication in this study was the cost to the payers and not necessarily the cost to the patients, and thus does not exactly reflect the reality of the patients’ cost burdens. Terbinafine is likely highly prescribed due to easy accessibility, low cost, generic availability, and excellent safety profiles and efficacies [19]. While fluconazole is used off-label for onychomycosis therapy in the United States (but approved for treatment of onychomycosis in Europe), it may also be frequently prescribed due to its broad-spectrum coverage and absorption that is independent of food or gastric pH (unlike itraconazole) [19]. In an analysis of oral fluconazole prescribing in the Medicare provider utilization and payment database (2014 to 2019), oral fluconazole prescriptions had an average annual growth rate of 8.7%, and total fluconazole spending increased by 18% overall [20]. 

Griseofulvin and itraconazole were the least commonly prescribed systemic medications, and, over the study period, griseofulvin and itraconazole prescriptions have decreased. Griseofulvin is now rarely used for onychomycosis treatment, most likely due to the longer duration of use (at least 6 months), higher risk of adverse events, and lower cure rate compared to other antifungals [19,20]. In a double-blind clinical trial of 89 patients with onychomycosis, 42% of patients had complete cure, and 84% had mycological cure with terbinafine compared to only 2% and 45%, respectively, with griseofulvin [19]. Additionally, in a double-blind parallel group study analyzing treatments for toenail onychomycosis, the frequency of side effects was lower with 250 mg per day terbinafine for 16 weeks (11%) than with 500 mg per day griseofulvin for 52 weeks (29%) [21]. In the aforementioned observational study of Medicare provider utilization and payment data analyzing onychomycosis antifungal prescription cost and trends in utilization by dermatologists, the use of itraconazole was 150-fold lower than the use of terbinafine, with claims peaking in 2014 and then decreasing by 0.9% annually, and with the total spending decreasing by 35.6% overall [18]. Itraconazole cost per supply day decreased by 7.4% during the study period [21]. Itraconazole is associated with numerous drug contraindications, which may result in lower prescription frequency and decreased prescribing over time [20].

We found that the most frequent topical medication prescribed was ciclopirox, and, over time, prescriptions have increased. Ciclopirox 8% nail lacquer was approved by the Food and Drug Administration (FDA) in 2003 for the treatment of fingernail and toenail onychomycosis. Similarly, in the aforementioned observational study of Medicare provider utilization and payment data, ciclopirox claims experienced an annual average growth rate of 8.7%, and total spending increased by 66.9% [18]. It may have been the more commonly prescribed topical in our study, due to it being FDA approved for a longer period of time than other topicals and its lower cost [19]. 

Our study also demonstrated that topical efinaconazole and topical tavaborole were less commonly prescribed than topical ciclopirox. They were both newer therapies approved in 2014 by the FDA for toenail onychomycosis. In the aforementioned observational study of Medicare provider utilization and payment data, efinaconazole claims peaked in 2015, then decreased by 5.6% annually, and the total cost and cost per supply day increased yearly by 3091% and 144%, respectively (2014 to 2018) [18]. Tavaborole claims decreased by 0.9% annually (2015 to 2018), but the tavaborole cost per supply day increased by 42.4% annually (2014 to 2018) [18]. Overall, efinaconazole and tavaborole were newer, less accessible, and more costly antifungal topical treatments (ciclopirox 6.6 mL, USD 23.45, compared to efinaconazole 4 mL, USD 577.36, or tavaborole 4 mL, USD 608.66) [19,22,23]. However, they have fewer potential severe adverse effects compared to systemic antifungals [24,25]. 

This study is subject to certain limitations. We included patients in this study based on the diagnostic codes listed above, and definitive diagnosis of onychomycosis by laboratory techniques, including KOH with microscopy, clipping with histopathology, fungal culture, and molecular biology techniques, including polymerase chain reaction, were not available in the All of Us database. This may result in other nail dystrophies that are not onychomycosis being included and analyzed in the onychomycosis population. In addition, due to lack of mycological confirmation, we may have also excluded bonafide onychomycosis cases. Moreover, this is a study based on information extrapolated from the All of Us database, and it is not an observational or experimental study.

In sum, our study found that age, obesity, tinea pedis, PVD, venous insufficiency, diabetes mellitus, and HIV are all significant risk factors for onychomycosis, consistent with prior research. Identifying and managing these risk factors is essential in preventing onychomycosis primary infections and recurrences. Moreover, our findings suggest that treatment trends for onychomycosis have emphasized efficacy while also considering patient convenience, accessibility, and affordability. As new antifungal medications and devices for treating onychomycosis will continue to emerge over time, keeping the patient at the center and prioritizing efficacy and accessibility are important factors to consider for achieving the best possible patient outcomes.

## Figures and Tables

**Table 1:** Demographics, comorbidities, and treatment courses of onychomycosis patients and case–controls in the All of Us research program.

	Onychomycosis Patients (*n* = 15,760)	Case Controls (*n* = 47,280)		
**Average age (SD)**	64.9 (12.9)	64.1 (13.1)		
Female	63.7 (13.0)	63.0 (13.2)		
Male	66.2 (12.6)	65.6 (12.9)		
Other	66.3 (12.2)	66.0 (11.7)		
**Sex, *n* (%)**				
Female	8535 (54.2)	26,147 (55.3)		
Male	6996 (44.4)	20,513 (43.4)		
Other	229 (1.5)	620 (1.3)		
**Race, *n* (%)**				
White	8325 (52.8)	25,556 (54.1)		
Black	3634 (23.0)	10,539 (22.3)		
Other	3801 (24.1)	11,185 (23.7)		
**Ethnicity, *n* (%)**				
Hispanic/Latino	2804 (17.8)	8306 (17.6)		
Non-Hispanic/Latino	12,956 (82.2)	38,974 (82.5)		
**Co-diagnoses, *n* (%)**			**Odds ratio**	***p*** -value *
Obesity	7311 (46.4)	11,934 (25.2)	2.59 [2.49–2.69]	<2.2 × 10^−16^
Tinea pedis	3394 (21.5)	1174 (2.5)	10.9 [10.1–11.6]	<2.2 × 10^−16^
Peripheral vascular disease	2266 (14.4)	2439 (5.2)	3.04 [2.86–3.24]	<2.2 × 10^−16^
Venous insufficiency	2111 (13.4)	2053 (4.3)	3.38 [3.15–3.59]	<2.2 × 10^−16^
Venous varices	890 (5.6)	1016 (2.1)	2.71 [2.47–2.97]	<2.2 × 10^−16^
Diabetes mellitus	888 (5.6)	831 (1.8)	3.28 [2.98–3.61]	<2.2 × 10^−16^
HIV	556 (3.5)	944 (2.0)	1.8 [1.61–2.00]	<2.2 × 10^−16^
Immunosuppression	33 (0.2)	73 (0.2)	1.37 [0.90–2.06]	0.21
Hyperhidrosis	<20 (<0.1) †	21 (0.04)	2.14 [1.1–4.15]	2.5 × 10^−2^
**Medication use, *n* (%)**			**95% CI**	
Oral				
Terbinafine	3287 (20.9)	N/A	20.2–21.5	
Fluconazole	2707 (17.1)	N/A	16.6–17.8	
Griseofulvin	171 (1.09)	N/A	0.93–1.26	
Itraconazole	167 (1.05)	N/A	0.91–1.24	
Topical				
Ciclopirox	1961 (12.4)	N/A	11.9–13.0	
Efinaconazole	403 (2.6)	N/A	2.31–2.81	
Tavaborole	54 (0.3)	N/A	0.26–0.45	
**Procedures, *n* (%)**			**95% CI**	
Debridement of nail(s)	3044 (19.3)	N/A	18.7–19.9	
Nail avulsion or removal	688 (4.4)	N/A	4.04–4.70	
Trimming of nail(s)	464 (2.9)	N/A	2.69–3.22	

CI: confidence interval; HIV: human immunodeficiency virus; N/A: not applicable; SD: standard deviation. * Benjamini–Hochberg adjusted *p*-values corresponding with a false discovery rate (FDR) <0.1. † Less than notation is required for compatibility with All of Us policies that help protect patient privacy.

**Table 2:** Age of patient when onychomycosis was diagnosed, divided by the number of patients in each age group with EHR data.

Decade of Life at First Diagnosis	Number of Onychomycosis Cases	Fraction *	95% CI
20–29	477	0.031	[0.028–0.034]
30–39	1290	0.040	[0.038–0.042]
40–49	2424	0.075	[0.073–0.078]
50–59	4371	0.105	[0.102–0.108]
60–69	4493	0.090	[0.088–0.093]
70–79	2255	0.060	[0.058–0.063]
80–89	357	0.029	[0.026–0.032]

* Number of onychomycosis patients/number of patients with EHR data of the same age.

**Table 3:** Antifungal medication prescription trends over time for patients with a diagnosis of onychomycosis in the All of Us research program.

Treatment	Spearman Correlation *	*p*-Value
Ciclopirox	0.182	0.0361
Efinaconazole	0.126	0.644
Fluconazole	0.665	2.44 × 10^−4^
Griseofulvin	−0.557	0.0131
Itraconazole	−0.681	3.32 × 10^−6^
Tavaborole	−0.663	0.0694
Terbinafine	−0.219	0.647

* Spearman correlation between fraction of treatments accounted for by each drug and year. Positive values suggest an increase in frequency of medication over time, whereas negative values suggest that the frequency of medication has decreased over time.

## Data Availability

The data that supports the findings of this study are available from the corresponding author Shari Lipner (shl9032@med.cornell.edu) upon request.

## Abbreviations

QoL	Quality of life
EHR	Electronic health record
FDR	False discovery rate
OR	Odds ratio
CI	Confidence interval
ICD	International Classification of Diseases
SNOMED	Systematized Nomenclature of Medicine
PVD	Peripheral vascular disease
HIV	Human immunodeficiency virus
PAD	Peripheral arterial disease
ABI	Ankle/Brachial Index
FDA	Food and Drug Administration
